# A quasi-monoenergetic short time duration compact proton source for probing high energy density states of matter

**DOI:** 10.1038/s41598-021-86234-x

**Published:** 2021-03-25

**Authors:** J. I. Apiñaniz, S. Malko, R. Fedosejevs, W. Cayzac, X. Vaisseau, D. de Luis, G. Gatti, C. McGuffey, M. Bailly-Grandvaux, K. Bhutwala, V. Ospina-Bohorquez, J. Balboa, J. J. Santos, D. Batani, F. Beg, L. Roso, J. A. Perez-Hernandez, L. Volpe

**Affiliations:** 1 Centro de Laseres Pulsados (CLPU), Parque Cientifico, 37185 Villamayor, Salamanca Spain; 2 Department of Electrical and Computing Engineering, University of Alberta, Edmonton, AB T6G 2V4 Canada; 3 University of Salamanca, Salamanca, Spain; 4 CEA, DAM, DIF, 91297 Arpajon, France; 5 CNRS, CEA, CELIA (Centre Lasers Intenses et Applications), UMR 5107, University of Bordeaux, 33405 Talence, France; 6 Center for Energy Research, University of California San Diego, La Jolla, CA 92093 USA; 7 Laser-Plasma Chair at the University of Salamanca, Salamanca, Spain; 8 Instituto Universitario Física Fundamental y Matemáticas, 37008 Salamanca, Spain

**Keywords:** Astronomy and astrophysics, Fluid dynamics, Nuclear physics, Plasma physics, Astronomy and planetary science, Energy science and technology, Physics

## Abstract

We report on the development of a highly directional, narrow energy band, short time duration proton beam operating at high repetition rate. The protons are generated with an ultrashort-pulse laser interacting with a solid target and converted to a pencil-like narrow-band beam using a compact magnet-based energy selector. We experimentally demonstrate the production of a proton beam with an energy of 500 keV and energy spread well below 10$\%$, and a pulse duration of 260 ps. The energy loss of this beam is measured in a 2 $\upmu$m thick solid Mylar target and found to be in good agreement with the theoretical predictions. The short time duration of the proton pulse makes it particularly well suited for applications involving the probing of highly transient plasma states produced in laser-matter interaction experiments. This proton source is particularly relevant for measurements of the proton stopping power in high energy density plasmas and warm dense matter.

## Introduction

During the last decades, multi-Terawatt and Petawatt laser facilities have become standard across the world^1^, allowing the study of new regimes of laser-plasma interaction. Short pulse lasers with intensities above 10$^{18}$ W/cm$^2$ have been widely used for the generation of compact, high brightness particle and radiation sources. One of the most commonly used techniques for proton generation is the mechanism known as Target Normal Sheath Acceleration (TNSA)^2,3^. It opens up the possibility to generate multi-MeV protons up to 100 MeV energy^4^ and ion beams with high brilliance and relatively short time duration at high repetition rate. The strongly localized energy deposition of ions in matter, due to the existence of the Bragg Peak, is an important characteristic for many applications, including proton therapy^5,6^, ion-induced isochoric heating of matter^7–10^, proton radiography^11,12^, alpha particle heating in inertial confinement fusion (ICF)^13,14^, the proton fast ignition approach to ICF^15^, and heavy ion fusion^16,17^.

The energy spectrum of laser-driven proton beams, however, is typically very broad in spectrum (tens of MeV), and divergent in transverse profile^2,3^. For this reason, many applications require processing of the proton beam^18–21^ to either modify the spatial profile (collimation and focusing) or their energy spectrum (narrow energy selection). In Ref.^18^ they propose the use of quadrupole lens to focus the proton beam for imaging purposes while in Ref.^19^ pulsed solenoids are used to perform effective focusing. A technique for micro-lensing and energy selection is proposed in Ref.^20^. In Ref.^21^ an approach dedicated just to the energy selection is shown, achieving bandwidths of tens of keV. The above mentioned techniques cannot offer a short time spread of the proton bunch, which is critical for particular applications where the time scale is below the nanosecond.

Here we report a novel design for such a proton energy selector and its experimental characterization performed at the Centro de Laseres Pulsados (CLPU) VEGA 2 laser facility^22^. The system allows one to select a bunch of laser generated protons with an energy spread of few percent. We present results of proton beams of E = 498 ± 4 keV with a minimum energy bandwidth $\Delta$E = 33 ± 4 keV at Full Width at Half Maximum (FWHM), which corresponds to $\Delta$E/E = 6 ± 0.75 $\%$. The selector is based on the use of a 1.2 T compact permanent dipole magnet, with a longitudinal dimension of $\sim$ 6 cm. The geometry allows a very close positioning of the selector to the proton source. The main features of this energy selector are the small energy bandwidth and the short time spread of the selected proton beam. These are both necessary properties for many applications where the probed target evolves in time or where the characteristic time of interaction is short and below the nanosecond time scale. A minimum time spread of 260 ± 15 ps is obtained in the setup described here. One of the applications demanding a short time spread of the proton beam is proton stopping power measurements in extreme states of matter^23–26^ that exist for a short time and whose parameters change on a sub-nanosecond time scale.

## Results

### Energy selector optimization

**Figure 1:**
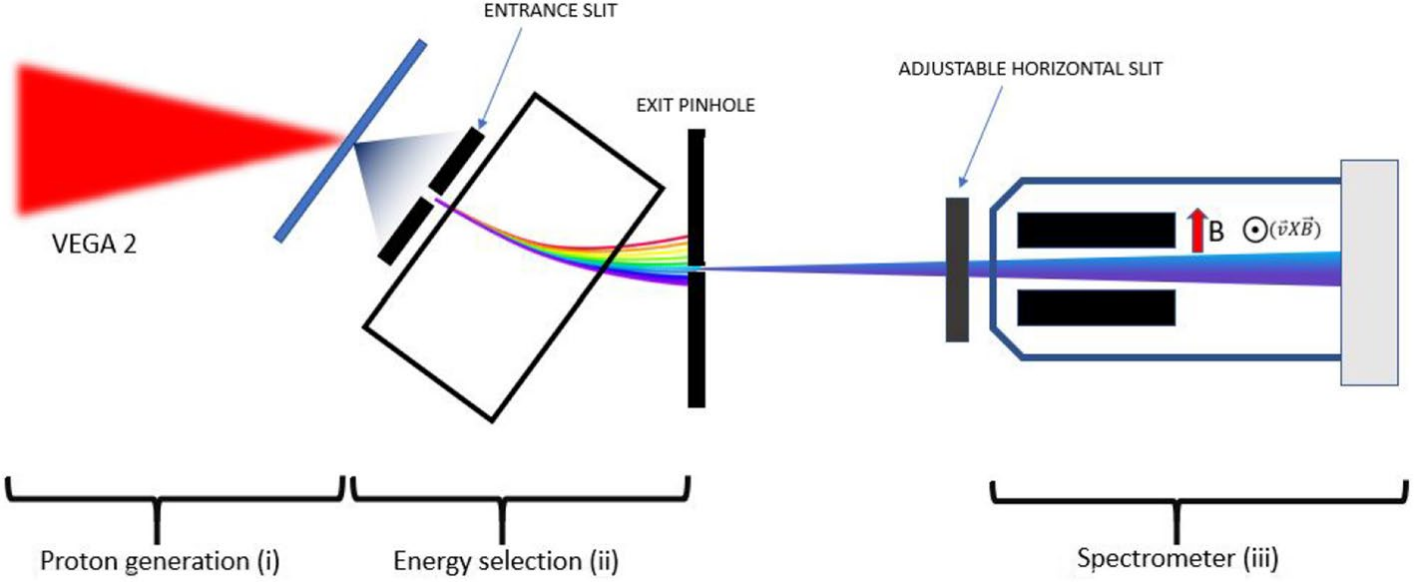
Scheme of the experimental setup. It can be divided into three stages: proton generation (i), energy selection using magnet based proton selector (ii) and proton energy measurement with the magnet spectrometer (iii).

The characterization of the proton energy selector was performed at the CLPU using the 30 fs, 200 TW system VEGA 2. The scheme of the experimental setup is shown in Fig. 1. The experimental scheme consists of three main stages: (i) the VEGA 2 laser is used to accelerate a broadband-spectrum proton beam through the TNSA mechanism, (ii) the energy selector selects out a proton bunch at a specific energy with narrow energy and angular spread, and (iii) the selected proton bunch is measured with a magnet-based spectrometer. A central proton energy of 500 keV was chosen for this setup. This projectile energy was used for subsequent measurements of the proton stopping power in warm dense matter (WDM), that will be presented in a future publication. The entrance slit and the exit pinhole of the selector are adjusted to optimize the proton flux and the energy bandwidth at the desired energy. The high repetition rate of the system allowed a statistical characterization of the proton energy selection.

**Figure 2:**
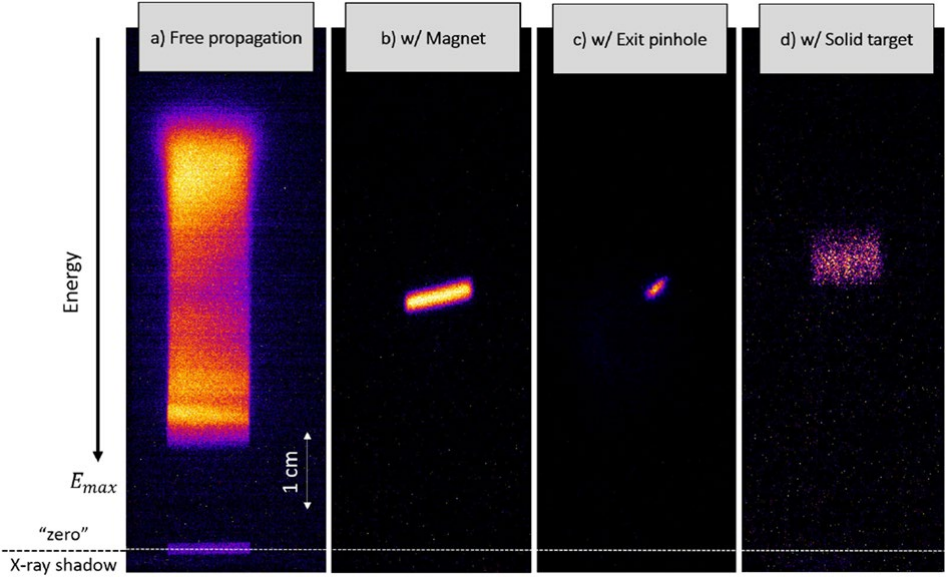
MCP traces in three configurations: (**a**) Full proton beam with no magnetic selector in (full TNSA spectrum). (**b**) Partial selection magnetic selector in with only the 20 $\upmu$m entrance slit. (**c**) Full selection with 20 $\upmu$m entrance slit and 20 $\upmu$m exit pinhole). (**d**) Selected beam (**c**) after passing through a solid 2 $\upmu$m Mylar foil.

**Figure 3:**
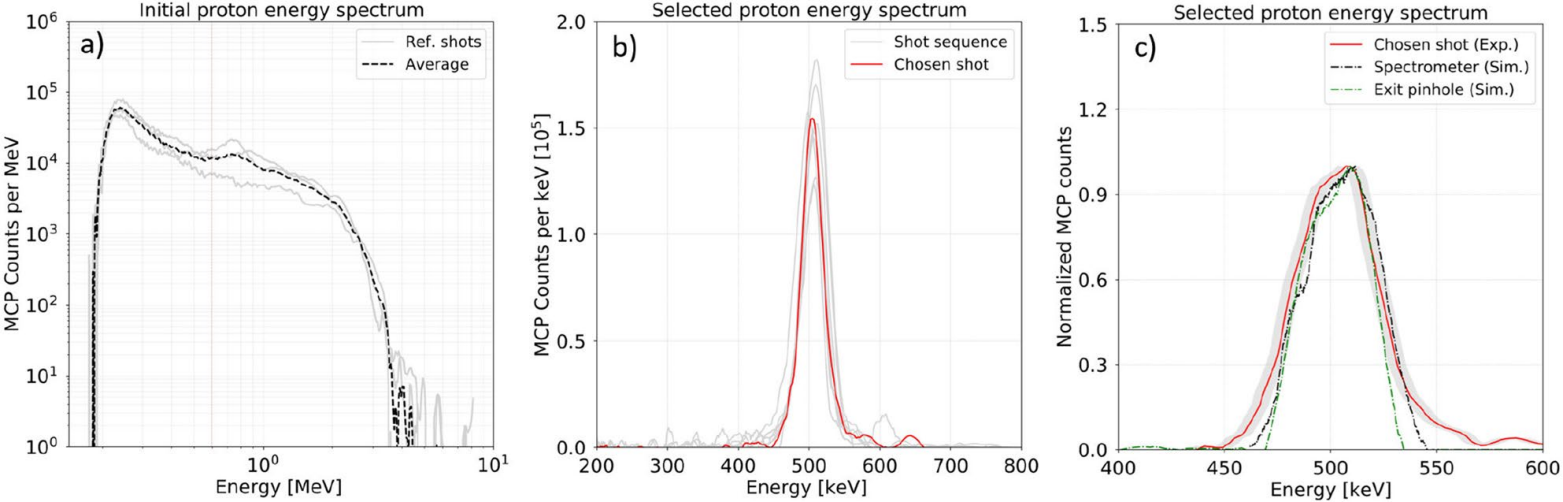
Spatial sampling of experimental spectra measured with magnet spectrometer. (**a**) Several example spectra obtained in configuration of no selection featuring reference initial proton spectrum with cut-off energy of $\sim$ 4 MeV (3000 V MCP Voltage). (**b**) The selected proton beam spectra (light-grey curves) obtained in full selection using 20 $\upmu$m entrance slit and 20 $\upmu$m exit pinhole (5000 V MCP Voltage). The chosen shot represents the typical spectrum (red solid curve). (**c**) Comparison of the chosen shot with synthetic proton spectrum obtained with MC FLUKA simulations at the exit pinhole (green dashed curve) and at spectrometer position (black dashed curve) in corresponding experimental geometry.

Figure 2 shows different traces as recorded in the spectrometer detector: Fig. 2a shows the original TNSA proton beam as measured without selection. In Fig. 2b the magnet selector was placed in and only the protons passing through the first entrance slit (20 $\upmu$m aperture) were entering the spectrometer. Figure 2c shows the full proton energy selection when the exit pinhole (20 $\upmu$m diameter size) after the magnet is in place. The corresponding energy spectra for the full TNSA proton beam (Fig. 2a) and the selected proton beam (Fig. 2c) are shown in Figs. 3a and b with examples of statistical comparison of the beam energy stability, while Fig. 3c shows the comparison between experimental data and simulations. The proton beam profile at the Micro-Channel-Plate (MCP) detector is the result of dispersion of the beam in the horizontal plane introduced by the magnetic selector (magnetic field oriented in vertical direction) with an additional spatial-modulated deflection induced by the Lorentz force in the magnetic spectrometer which results is an apparent rotation of the trace. Finally, Fig. 2d shows the pencil-like proton beam after passing through a 2 $\upmu$m Mylar solid foil that will be discussed in detail in the following section.

The selection of the proton beam of $\sim$ 500 keV energy was performed by using various apertures for entrance slit (20 and 50 $\upmu$m) and exit pinhole (10–200 $\upmu$m). The Table 1 displays the proton central energy and the energy bandwidth (FWHM) measured in each configuration.

**Table 1:** The energy bandwidth of selected $\sim$ 500 keV proton beam measured for each energy selector configuration of entrance slit and exit pinhole.

Entrance slit	Exit pinhole/slit	Central energy (keV)	Bandwidth (keV)
50 $\upmu$m slit	200 $\upmu$m slit	501 ± 8	79 ± 8
50 $\upmu$m slit	20 $\upmu$m pinhole	510 ± 4	54 ± 4
20 $\upmu$m slit	20 $\upmu$m pinhole	498 ± 4	44 ± 4
20 $\upmu$m slit	10 $\upmu$m pinhole	497 ± 4	33 ± 4

The values of the central energy and bandwidth presented in Table 1 show the average values of the energy bandwidth over N shots. The total error is estimated as $\sigma _{tot} =\sqrt{\sigma _{stat}^{2}+ \sigma _{sys}^2}$, where $\sigma _{stat} = \sigma /\sqrt{N}$ is a statistical error with $\sigma$ = 12 keV and N = 20 and $\sigma _{sys}\approx \pm$ 2.5 keV is a systematic error coming from the uncertainty in the vertical position of the measured proton signal with respect to the zero-deflection point at the MCP detector. The selected proton beam spectrum shown in Fig. 3b features a central energy of $E_{c}$= 498 ± 4 keV with an energy bandwidth of $\Delta _{E_c}$ = 44 ± 4 keV (FWHM). Such error on the central energy and energy bandwidth suggests that the selected proton energy and energy bandwidth have low sensitivity to the laser shot-to-shot instability (pointing stability $\sim$ 12 $\upmu$m, energy variation $\sim$ 3 $\%$). The configuration using the 20 $\upmu$m entrance slit and 20 $\upmu$m pinhole has an optimal performance in terms of both a small energy bandwidth and a reasonable proton flux ($\sim$ 1500 detected protons at the MCP). The absolute number of protons is estimated using an MCP calibration made with proton energies $< 1\, \hbox {MeV}$^27^. The selected proton beam has also been characterized in terms of spatial size for this configuration. The proton beam spot of 50 $\upmu$m diameter has been measured 0.9 cm away from the exit pinhole using Radiochromic Film (RCF) placed on the proton propagation axis after selection. The result agrees well with the observed proton signal size at MCP spectrometer $\sim$ 1 mm (60 cm from the exit of pinhole).

### Estimation of initial proton source size

It is well known that laser produced proton sources can exhibit initial transverse dimension much bigger than the laser spot^28,29^. The source size can range to several hundreds of microns for the lowest proton energies. Under these conditions, proton sources cannot be assumed to be point-like and the effect of the proton divergence (maximum angular spread collected by apertures) is mainly controlled by the initial source size and the distances to the exit pinhole and to the MCP of the spectrometer. The angular spread of the proton beam plays a double role. On the one hand, it creates a geometrical magnification of the beam at the MCP detector. On the other hand it is responsible for the minimum achievable bandwidth, i.e. the more angular spread, the more spread in the energy of the trajectories that can pass through the selector.

**Figure 4:**
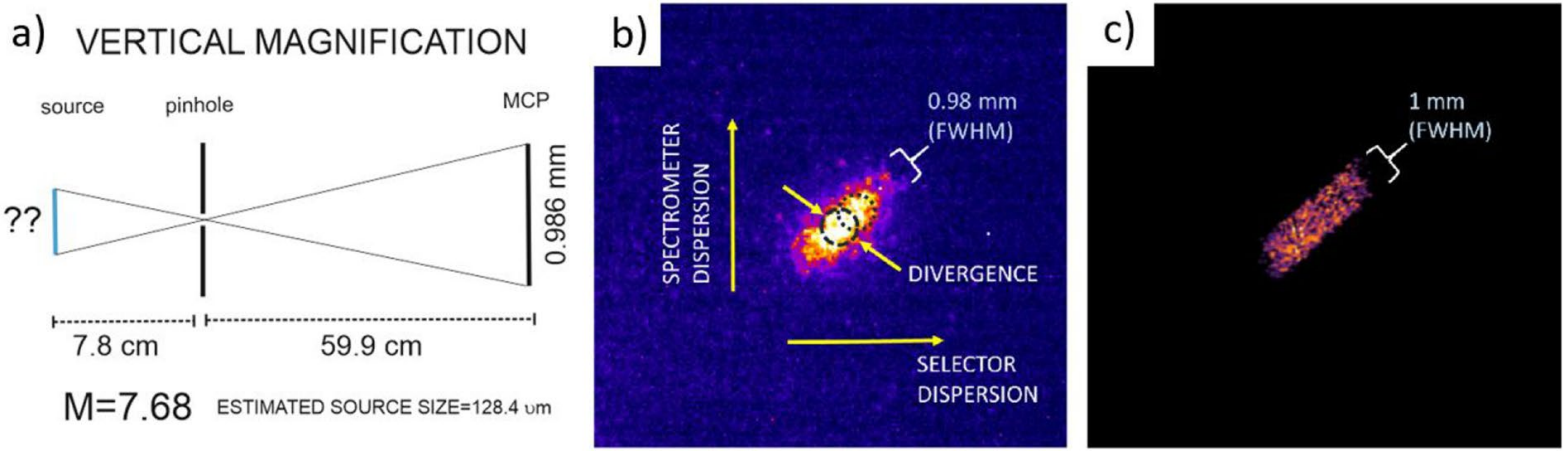
(**a**) Sketch of the point like pinhole magnification system. (**b**) Typical proton signal on MCP obtained with configuration of 20 $\upmu$m slit and 20 $\upmu$m pinhole that corresponds to 44 keV energy bandwidth (FWHM) and proton trace width of $\sim$ 0.98 mm. (**c**) Proton signal on MCP obtained with FLUKA simulation reproducing an experimental result of energy bandwidth of 44 keV (FWHM) and width of $\sim$ 1 mm using initial source size of 150 $\upmu$m.

Experimental conditions did not permit us to have a direct measurement of the initial proton source size at the rear side of the target. However one can deduce the proton source size by using a simple model as shown in Fig. 4a. The magnified source size is calculated from the width of the MCP proton trace as is appearing on the screen after passing through an ideal point-like pinhole. The magnification factor is estimated using the distance from the proton source to the exit pinhole, the distance from this pinhole to the MCP detector and the measured trace width on the detector which is $\sim$ 0.98 mm (FWHM) as shown in Fig. 4b. The dimension and the orientation of the trace depend both on the two magnetic deflections. The first is inside the selector (horizontal dispersion) and the other is inside the spectrometer (vertical dispersion). The width of the trace represents a vertical image of the proton source. The final magnification is calculated to be 7.7 and implies an initial proton source size of around 150 $\upmu$m, which is in agreement with simulations performed using the Monte Carlo (MC) code FLUKA^30^ shown in Fig. 4c.

### Modelling of the minimum selected bandwidth

**Figure 5:**
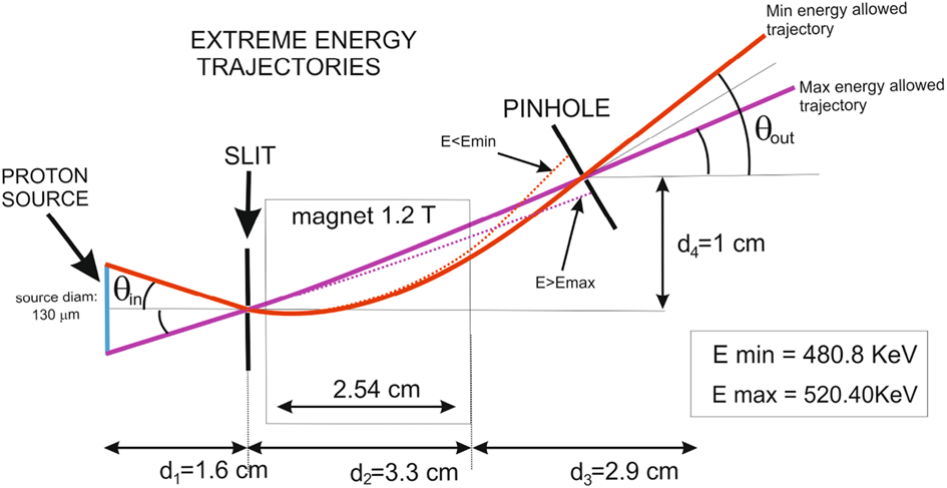
Scheme of maximum and minimum allowed energies due to horizontal divergence. Estimated values using simple gyroradius model in a square B field are given.

Once the initial transverse size is estimated we can also describe the effect of the beam angular spread on the bandwidth. It can be modeled in a simple way as shown in Fig. 5. This simple model shows the effect of the source size on the energy bandwidth at the exit pinhole ($\Delta E_{P}$). Minimum and maximum energy allowed trajectories are graphically shown and estimated as a function of incidence angle $\theta _{in}$ and energy using a simple 2D model of proton gyro-radius in a flat-top B-Field.

Beyond this simple model, Monte Carlo simulations (FLUKA) were employed to simulate the full trajectories in 3 dimensions. This allowed us to study the effect of the proton source size on the energy bandwidth at the exit pinhole $\Delta E_{P}$ compared to the one measured at the spectrometer position $\Delta E_{S}$ as shown in Table 2. The simulation results suggest that there is no major effect of angular spread (proton source magnification) on the measurement of the energy bandwidth at the spectrometer ($\Delta E_{P} \approx$ $\Delta E_{S}$). The example of the proton trace at MCP shown in Fig. 4b has been reproduced with FLUKA simulations by using proton source size of 150 $\upmu$m that would yield in proton trace of 1 mm width (Fig. 4c).

**Table 2:** Selected $\sim$ 500 keV proton beam bandwidth (FWHM) and spatial size as a function of the proton source size obtained with FLUKA MC simulations. The best fit to the experimental result is highlighted.

Source size ($\upmu$m)	Width (mm)	$\Delta E_{P}$ (keV)	$\Delta E_{S}$ (keV)
100	0.72	27.5	33.8
110	0.77	32.1	32.5
120	0.88	34.4	35
130	0.96	36.9	39
**150**	**1.00**	**41.9**	**43.6**
180	1.19	51.2	54

Moreover, results are also in good agreement by considering a source size of 150 $\upmu$m previously discussed for pinhole imaging magnification model. The simulations also agree with the simple model prediction of energy bandwidth $\Delta E_{P}$ scaling as a function of the proton source size.

The simulation results indicate that in order to further decrease the energy bandwidth one would need to reduce the experimental proton source size using additional techniques such as wire-targets^31^, self-generated magnetic fields^32^ or mass-limited targets^33^ or by introducing an additional pinhole in the magnet selector with a consequent reduction of the number of selected protons.

When the proton beam is used for proton stopping power measurements, the final output is completely dominated by the proton multiple scattering in the sample, magnifying the final transverse beam size. Despite the increase of scattering, the final beam shows a high degree of spatial and energy uniformity and this permits a measurement in a reduced area of the beam without losing energy precision. In particular, it is possible to use a thin slit (perpendicular to the Lorentz force direction) to increase the final spectral resolution.

### Energy loss measurement in solid target

We used the described setup to measure the energy loss of a 510 keV proton beam in a sample solid target of 2 $\upmu$m Mylar coated with 40 nm Aluminum, having a total areal density of 240 ± 10 $\%$ $\upmu$g/cm$^{2}$. The target has been placed on the proton beam axis 0.9 cm away from the exit pinhole of selector. The experimental proton signal on the MCP detector after passing through the foil is shown on the Fig. 2d. One can see that the signal has been broadened and shifted towards lower energy. The modification of the proton beam profile is directly related to the proton beam straggling and scattering in the solid target^12,34^. The proton beam scattering complicates the estimation of the central energy of the proton beam at the MCP detector and thus increases error of the energy loss measurement. However the insertion of an additional horizontal slit in front of the spectrometer reduces the scattering effect on the MCP detector and allows for a more precise measurement of the central energy. The multiple scattering of protons in matter can be considered by estimating the mean scattering angle $\theta = \sqrt{A\,(\text {g/cm}^{3})}/E\, (\text {MeV})$^12^ for a pencil-like proton beam of 510 keV energy entering a 240 $\upmu$g/cm$^{2}$ areal density target, which gives $\theta$ $\sim$ 3$^\circ$. Such a scattering angle produces a beam much larger than the entrance of the spectrometer. The FLUKA simulations predict that a partial collection of the protons increases the measurement uncertainty in the central energy by up to 3.5 keV.

**Figure 6:**
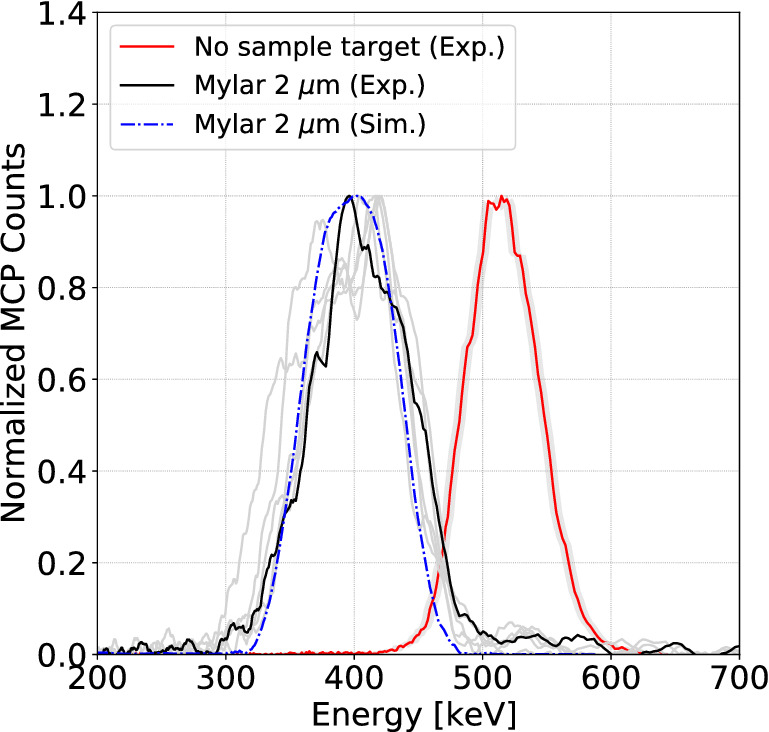
The experimental initial proton spectrum (red curve) and the downshifted proton spectrum after passing through 2 $\upmu$m Mylar coated with 40 nm Aluminum (black curve). The additional spectra (grey curves) are shown to demonstrate the repetitive behaviour. The simulated downshifted spectrum (dashed blue curve) is obtained with FLUKA MC simulation.

Figure 6 shows the experimental proton spectrum with central energy of 510 ± 4 keV obtained with 50 $\upmu$m entrance slit and 20 $\upmu$m exit slit and the energy spectra of the proton beam after passing through the solid target. The downshifted experimental spectra are also compared with the downshifted spectrum at the spectrometer obtained using FLUKA MC simulations. The measured energy loss of 510 keV protons in the sample target is 106 ± 7 keV. The total error consists of $\sqrt{\sigma ^2_{stat}+\sigma ^2_{sys}}$, where $\sigma _{stat} = \pm$ 6 keV and the systematic error is $\sigma _{sys} = \pm$ 3.5 keV coming from the aforementioned partial collection of protons. The measurement agrees with the value of 107 ± 11 keV predicted by the SRIM code^35^, which includes the energy loss error due to the uncertainty in the sample areal density as specified by the manufacturer. The results of the energy loss are very repetitive and in good agreement with SRIM while the discrepancy of 106 ± 7 keV indicates that the real sample areal density is quite close to its nominal value.

### Simulations

**Figure 7:**
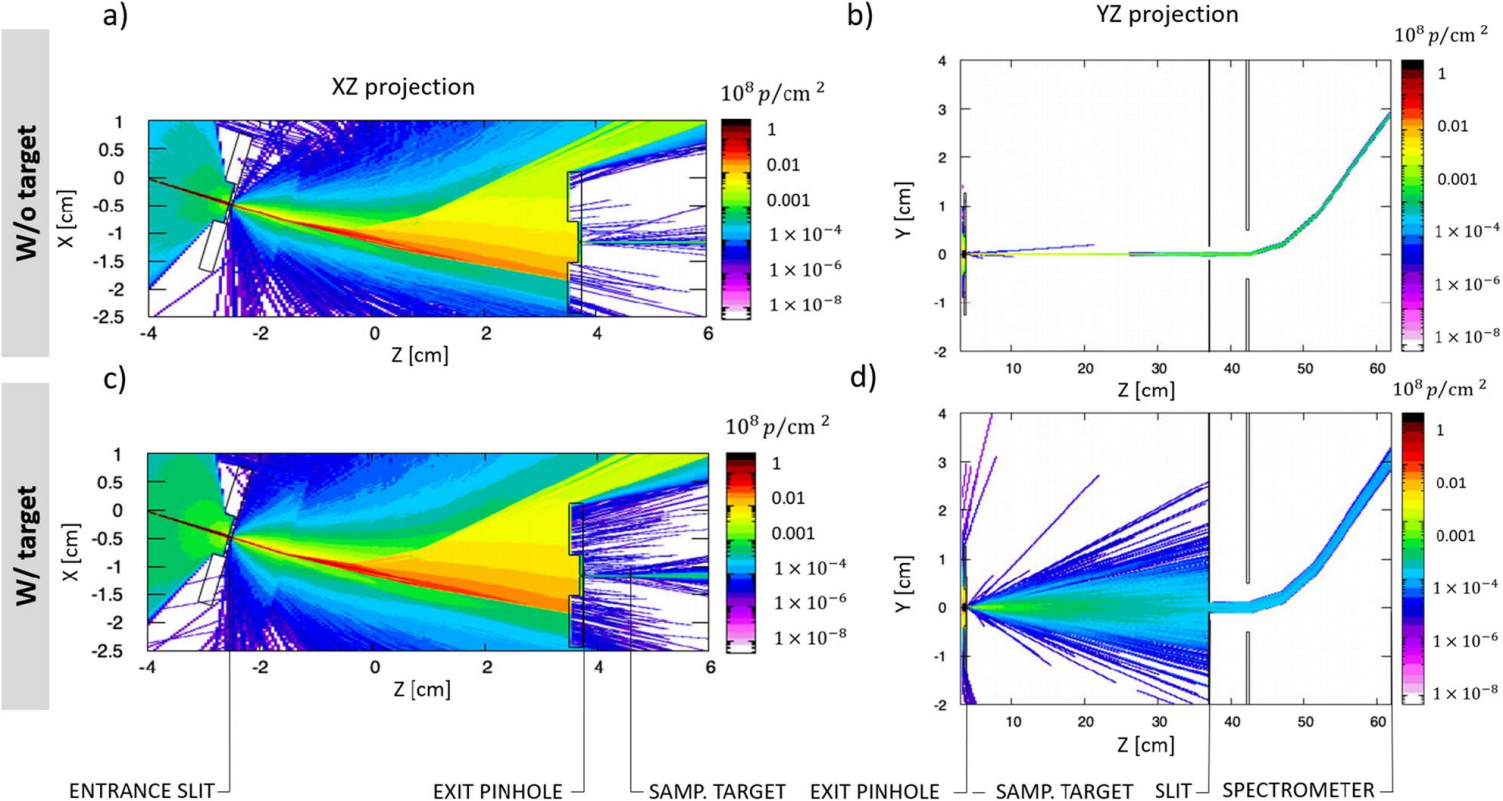
Monte-Carlo simulations performed with the FLUKA MC code. (**a**) The protons enter through the entrance slit, are deflected by the $B_{y} = 1.2$ T magnetic field, and 500 keV protons are selected by the exit pinhole. (**b**) Propagation of protons and deflection by the $B_{x}=0.2$ T magnetic field of MCP spectrometer. (**c**) and (**d**) Equivalent simulations including a 2 $\upmu$m Mylar target. Scattering in the target increases the proton divergence and only part of the beam is sampled by the entrance pinhole of the spectrometer.

The experimental results were modeled with 3D Monte-Carlo simulations using the FLUKA code^30,36^. Figure 7a shows the proton fluence in the energy selector for the experimental configuration of a 20 $\upmu$m entrance slit and 20 $\upmu$m exit pinhole. Figure 7b presents the propagation of the selected $\sim$ 500 keV proton beam from the pinhole to the MCP screen, where the proton beam is deflected vertically by the magnetic field of the spectrometer.

A synthetic spectrum at the exit of the pinhole was recorded using an absolute energy detector with a resolution of 2 $\times$ $10^{-4}$ MeV integrated over the solid angle as shown in Fig. 3c. A synthetic image of the proton signal on the MCP screen with a resolution of 7 $\upmu$m (Fig. 4b) was used for comparison with the experimental image on MCP.

Figure 3c shows a comparison between the simulated spectra and the experimental spectrum on the MCP (solid red curve). The obtained simulated spectra are recorded at the exit of the pinhole by the absolute detector (dashed green curve) and at the spectrometer position (dashed black curve) considering a source size of 150 $\upmu$m. The simulation results show a small effect in the readout of final spectra induced by the proton divergence that only affects the lower part of the distribution modifying its wings. This indicates that the measurement of energy bandwidth (FWHM) at the exit of the pinhole $\Delta E_{P}$ is in agreement with the energy bandwidth on the spectrometer $\Delta E_{S}$ within 3 keV. A parametric study of the spatial size and the energy bandwidth of the selected proton beam as a function of the initial real proton source size has been performed in order to find the best fit to the typical experimental signal with an energy bandwidth of 44 keV and proton trace of $\sim$ 1 mm thickness. We finally conclude that the proton beam angular spread has a small and controllable effect on the final bandwidth readout in the spectrometer and this effect can be easily mitigated by moving the magnet spectrometer closer to the exit pinhole and by introducing a thin slit perpendicular to the Lorentz force for the case of dominant Multiple Scattering in the stopping power measurement. In order to calculate the time spread of the corresponding selected proton beam in FLUKA, an artificial time of flight calculation was implemented, assuming $t_{0}$ = 0 at the position of the proton source. It was found that the selected beam of 500 keV central energy and 44 keV ± 4 bandwidth leaves the exit pinhole at 8.8 ns with a time spread of 360 ± 15 ps (FWHM), while the selected proton beam with 33 keV ± 4 bandwidth obtained with 10 $\upmu$m exit pinhole has a time spread of 260 ± 15 ps (FWHM). It is a minimum achieved time spread in the scope of this work.

Simulations of the proton beam propagation after passing though the solid target have been performed in order to study the scattering effect and its mitigation for the proton stopping power measurements. The Fig. 7c,d shows the equivalent simulation of proton energy selection (Fig. 7a,b) including sample target of 2 $\upmu$m Mylar coated with 40 nm Al. The sample target placed 0.9 cm away from the exit pinhole produces a scattering effect that dominates over the initial proton divergence. The scattered beam is artificially reduced by the 2 mm horizontal slit located in front of the 1 cm entrance of the spectrometer as shown in Fig. 7d. The slit decreases the number of entering particles drastically, however it reduces the size of the scattered beam on the MCP, allowing a precise estimation of the peak energy. Such sampling of the proton beam yields in the error of 3.5 keV calculated by the comparison of absolute downshifted spectra obtained with full and partial collection of the protons at the entrance of the spectrometer.

In summary, we present a new adjustable platform for proton energy selection from laser-plasma sources that has been designed, characterized and experimentally tested by measuring the proton stopping power in a solid plastic target. The novel design produces a narrow-band proton beam with a short time spread, as is required for numerous applications, and in particular, proton stopping power measurements in transient states of matter. After optimization, the selection of proton projectiles of 498 ± 4 keV central energy with an energy bandwidth of 44 ± 4 keV (FWHM) and a time spread of 360 ± 15 ps (FWHM) at the exit pinhole with the required signal level has been demonstrated using 20 $\upmu$m slit and 20 $\upmu$m pinhole apertures in the energy selector. Different configurations of the proton energy selector have been experimentally characterized in terms of the proton signal intensity and energy bandwidth. The energy bandwidth was gradually reduced from 79 keV to 33 keV by using pinholes of smaller sizes from 200 $\upmu$m to 10 $\upmu$m with a time spread ranging from 630 ps to 260 ps. Numerical simulations performed with FLUKA have been used to interpret the experimental results and demonstrate the effect of the proton source size on the intrinsic bandwidth of the selected proton beam and on the shape of the signal on the MCP. As a demonstration of feasibility of the platform, we also show the energy loss measurement of the selected proton beam in a 2 $\upmu$m thick plastic foil that is in good agreement with theoretical predictions. It is expected that the platform can serve as a short pulse tunable source of protons for a variety of applications in the future.

The developed energy selector reported here opens up various opportunities for proton stopping power experiments in transient states of matter like WDM. In particular, by allowing probing proton energies of few hundred keV, the described system gives access to stopping measurements at low $v_{p}/v_{th}$ in WDM, where $v_{p}$ is the projectile velocity and $v_{th}$ is the thermal velocity of plasma electrons. This low-velocity stopping regime, close to the Bragg peak, has not been experimentally investigated until now for coupled and degenerate target conditions. The only stopping measurements in WDM reported so far were performed at a high projectile energy corresponding to $v_{p}/v_{th}$ > 10^25^.

At CLPU, a WDM sample can be generated by a second femtosecond laser pulse with intensities of $10^{15} - 10^{16}$ W/cm$^{2}$, generating electron temperatures of 10 - 15 eV over hundreds of ps expansion time. By probing these WDM conditions with a selected proton beam of 500 keV energy, velocity ratios as low as $v_{p}$/$v_{th}$ $\sim$ 3 can be achieved^26^. By selecting 100 - 300 keV protons, even lower $v_{p}$/$v_{th}$ ratios approaching unity can be reached, probing the Bragg Peak of the projectile-target interaction. However, the scattering effect needs to be taken into account for these lowest velocity cases. The energy selector can then be coupled with proton focusing systems that allow the collection of all the protons scattered from the WDM target. Therefore, our work opens up a new unexplored regime for ion stopping studies.

Another application of interest is a reverse convolution of plasma temperature and density by using the energy loss itself for a diagnostic purpose. One can use the selector to produce 1 - 2 MeV protons, that would have much shorter time spread of 10 - 20 ps for probing plasma and extreme states of matter. The energy loss at such high projectile velocity is well-established, which provides a unique opportunity for precisely estimating the plasma conditions.

## Methods

### Experimental setup

The basic characteristics of VEGA II and its main beam transport system have been explained in detail elsewhere^22^. In order to accelerate protons via the TNSA mechanism, the VEGA 2 laser pulse is transported and focused by an F/13 (F = 130 cm) parabolic mirror onto a 3 $\upmu$m thick aluminium foil at a 14.5$^\circ$ incidence angle within a 20 $\upmu$m focal spot (FWHM), yielding an intensity on target of $\sim 10^{19}$ $\text {W}/\text {cm}^{2}$. To provide fast target replacement after each laser shot, a motorized sandwich target holder with a matrix of 45 $\times$ 45 apertures of 800 $\upmu$m diameter was used. The second stage of the proton energy selection is based on energy dispersion by a 1.2 T permanent magnet with adjustable entrance and exit apertures to control the energy bandwidth of the proton beam. The selected beam characterization is done by means of a magnetic spectrometer (0.2 T dipole magnet) coupled with a phosphor MCP detector and an imaging system, indicated as a third stage. Selected beam spectra were recorded at high repetition rate (0.1 Hz) with an energy resolution of 2 keV per pixel at 500 keV. The energy selection and its measurement are explained in detail in the following sub-sections.

### Energy selector

**Figure 8:**
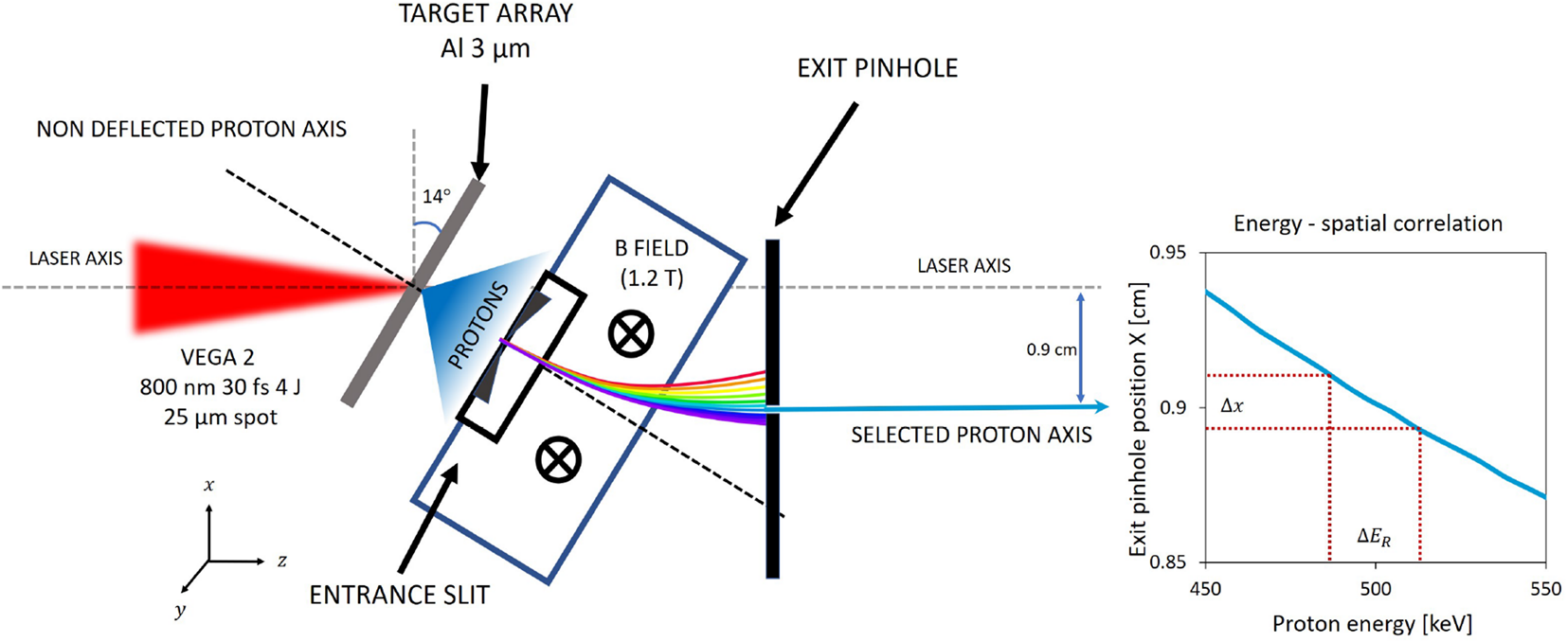
Scheme of the energy selection of 0.5 MeV proton beam with the energy spatial correlation.

The proton selector shown in Fig. 8 was designed to work with proton energies < 5 MeV and in particular as an adjustable platform for proton stopping power measurements. The system is designed in order to optimize the energy bandwidth and the time spread of the selected beam as a function of the initial beam divergence and spectrum. The proton energy selection is based on a magnetic selector working scheme with a dipole for horizontal deflection and two apertures - one before the magnet (entrance slit) and the other at the exit of the magnet (exit pinhole).

The entrance slit is directly attached to the dipole magnet yoke, located 1.6 cm from the proton source. It reduces the horizontal acceptance of the laser-driven TNSA proton beam which is characterized by a cone with an opening half-angle around 20$^{\circ }$ and selects a narrow pencil-like proton beam to enter the magnetic field zone. The rectangular slit is exchangeable to allow the selection of different apertures of 20 $\upmu$m and 50 $\upmu$m width and 3 mm height. The slits are from Thorlabs and the material is 50 $\upmu$m thick stainless steel 300. This selected beam then enters the field zone and is dispersed in the horizontal plane according to its energy.

The exit pinhole is located 1 cm after the exit of the magnet for the selection of a narrow bandwidth of proton energies of 500 keV as shown in Fig. 3b. Other energies can be selected by sliding the pinhole holder horizontally. The set of 10, 20, 200 $\upmu$m diameter pinholes is mounted in a motorized holder for fast change between pinholes of different diameters. Using a pinhole as the second filter instead of another slit allows a vertical control of the selected beam tilt angle. For these reasons, the exit pinhole is motorized in the horizontal direction (to allow the change of the selected energy) and in the vertical direction (to allow a tilt control and a pinhole exchange).

**Figure 9:**
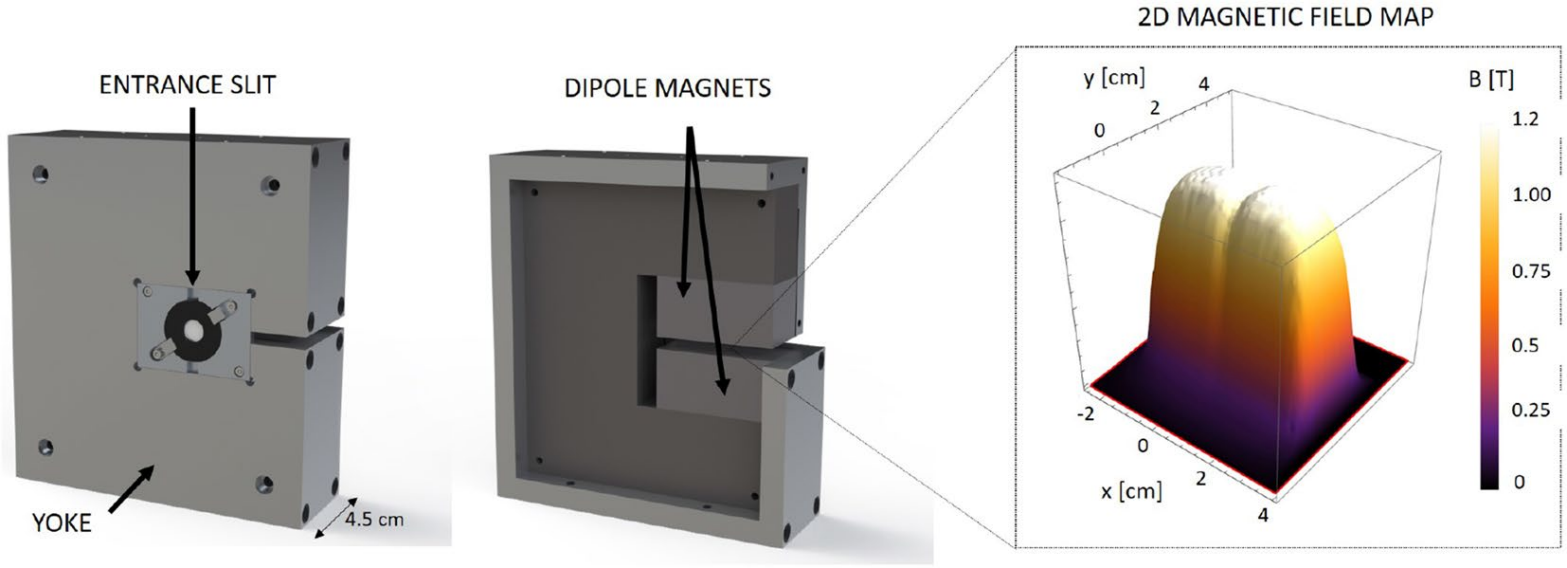
The design of magnet with entrance slit for the energy selector. 2D plot of the magnetic field generated by the dipole magnet.

The magnet for the energy selector was designed as a part of an internal project of CLPU on the development of high repetition rate diagnostics and secondary sources. It consists of a magnetic alloy yoke and two sets of 2.5 cm permanent magnets separated by a 5 mm gap in order to provide a constant magnetic field across a volume of 50.8 $\times$ 25.4 $\times$ 5 mm$^3$, as shown in Fig. 9. The 2D magnetic field map is also shown in Fig. 9. It was measured with a Hall effect Gaussmeter probe (Lakeshore) with a 1.02 mm active area over a range of 55 mm. Since protons in the 0.1 to 1 MeV range are easily deflected, step-like geometry fields (high squareness) are important to avoid proton deflection at long distances before and after the gap. The selector magnet is motorized in the transverse direction to provide an in-out movement for the acquisition of full TNSA spectra for reference purposes when put out. This motor axis was also used for the precise adjustment of the magnet position during the runs, and the vertical adjustment was manual.

The selected proton beam energy bandwidth is determined by the entrance slit and the exit pinhole. Smaller apertures and bigger separations between the entrance slit and the exit pinhole can provide smaller bandwidths but they also increase the time spread and decrease the number of protons. Therefore one should consider a trade-off between the desired low bandwidth and small time spread and the minimum required proton flux for application purposes. The proton pulse, when generated, has a few ps time duration^37^ but then it acquires time dispersion due to the different velocities of the protons in the bandwidth. Such time spread was limited to few hundreds of ps by reducing the total proton path length to 7.8 cm. The designed energy selector is compact with a length of 6.2 cm along the proton propagation axis and it can be located as close as needed to the proton source.

### Spectrometer

**Figure 10:**
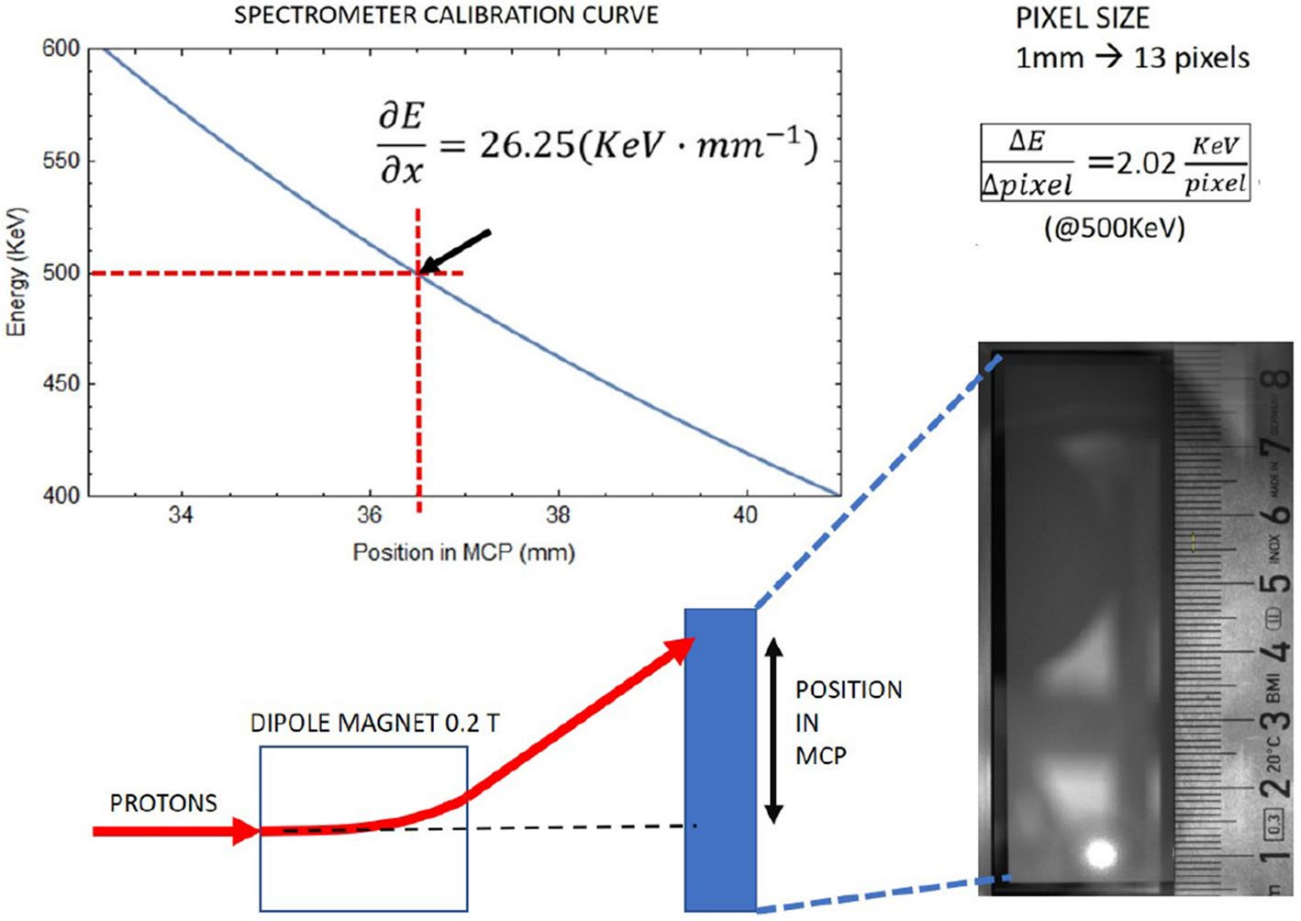
Sketch of the spectrometer. Simple expression of dispersion is given as a guide for the reader using a constant magnetic field gyroradius method.

A magnetic spectrometer was used to measure the central energy, the bandwidth and the profile of the proton beam. Figure 10 shows the deflection as a function of the magnetic field and the setup of the spectrometer.

The spectrometer was positioned 39 cm away from the exit pinhole of the proton energy selector along the propagation axis (Fig. 1). It consisted of a magnetic dipole generating a 0.2 T horizontal magnetic field 10.4 cm in length, and a detector composed of a Multichannel-Plate (MCP) coupled with a phosphor screen located 10 cm away from the end of the magnet and imaged onto a CCD camera. The spectrometer magnet yoke and the MCP were enclosed in a lead and Teflon shielding structure to protect them from the hard X-ray background. The clear aperture of the spectrometer is defined by a 1 cm diameter entrance hole. A horizontal slit of 1 mm aperture was located outside the shielding just in front of the entrance aperture. It ensured that only protons in the horizontal plane of the experiment enter the spectrometer, i.e. it set the “zero height” (zero deflection) position of the spectrometer. The horizontal slit was motorized in and out in order to reference the spectrometer in case of misalignment. This “zero” position was located in the MCP image using the shadowgraphic X-ray image of the slit observed from the proton source plasma with the magnet removed (Fig. 2a) shows an example).

Figure 10 shows the spectrometer calibration curve of energy versus position in the range of interest. The magnetic field map was scanned with the same Hall effect probe used for the selector magnet mapping. With the geometry and the fields of the spectrometer the energy resolution at 500 keV is 2.02 keV per pixel of the image.

### Numerical simulations

In the example simulation setup, 6$\times$ $10^{8}$ primary particles sources were used with an initial spectrum fitted to the TNSA experimental spectrum showed in Fig. 3a up to 2.5 MeV energy, an homogeneous angular distribution spread over 30 mrad and a circular source diameter of 150 $\upmu$m. The simulation box corresponded to the experimental geometry of the energy selector and the magnet spectrometer. A uniform selector magnetic field of $B_{y}$= 1.2 T was used in the simulation while the magnetic field of spectrometer was $B_{x}$=0.2 T.
